# Diet Quality Is Associated with Glucose Regulation in a Cohort of Young Adults

**DOI:** 10.3390/nu14183734

**Published:** 2022-09-10

**Authors:** Elizabeth Costello, Jesse Goodrich, William B. Patterson, Sarah Rock, Yiping Li, Brittney Baumert, Frank Gilliland, Michael I. Goran, Zhanghua Chen, Tanya L. Alderete, David V. Conti, Leda Chatzi

**Affiliations:** 1 Department of Population and Public Health Sciences, University of Southern California, Los Angeles, CA 90032, USA; 2 Department of Integrative Physiology, University of Colorado Boulder, Boulder, CO 80309, USA; 3 Department of Pediatrics, The Saban Research Institute, Children’s Hospital Los Angeles, Los Angeles, CA 90027, USA

**Keywords:** diet quality, dietary patterns, type 2 diabetes, prediabetes, obesity, body composition, young adults

## Abstract

Young-onset type 2 diabetes and prediabetes is a growing epidemic. Poor diet is a known risk factor for T2D in older adults, but the contribution of diet to risk factors for T2D is not well-described in youth. Our objective was to examine the relationship of diet quality with prediabetes, glucose regulation, and adiposity in young adults. A cohort of young adults (*n* = 155, age 17–22) was examined between 2014–2018, and 89 underwent a follow-up visit from 2020–2022. At each visit, participants completed diet and body composition assessments and an oral glucose tolerance test. Adherence to four dietary patterns was assessed: Dietary Approaches to Stop Hypertension (DASH), Healthy Eating Index (HEI), Mediterranean diet, and Diet Inflammatory Index (DII). Regression analyses were used to determine adjusted associations of diet with risk for prediabetes and adiposity. Each one-point increase in DASH or HEI scores between visits reduced the risk for prediabetes at follow-up by 64% (OR, 95% CI: 0.36, 0.17–0.68) and 9% (OR, 95% CI: 0.91, 0.85–0.96), respectively. The DASH diet was inversely associated with adiposity, while DII was positively associated with adiposity. In summary, positive changes in HEI and DASH scores were associated with reduced risk for prediabetes in young adults.

## 1. Introduction

The prevalence of prediabetes, a condition where blood glucose levels are elevated but below diagnostic cut-offs for type 2 diabetes (T2D) [1], is increasing in adolescents and young adults in the United States (U.S.) [2,3]. Prediabetes greatly increases the risk for T2D [4]; therefore, T2D incidence is also increasing in the U.S., following a similar trend [5]. This trend is of considerable concern because T2D is often more aggressive in youth than in older adults and is associated with higher rates of complications, more comorbidities, and higher mortality risk [6,7]. Disparities also exist in T2D risk, with Hispanics and other racial or ethnic minorities at higher risk compared to non-Hispanic Whites [5,7,8]. Lifestyle is a source of modifiable risk factors frequently targeted in preventive measures [1,9], of which diet is especially important.

Depending on quality, diet may be either protective against or a risk factor for prediabetes and T2D [10,11,12]. Healthy dietary patterns high in fruits, vegetables, and whole grains and low in sodium, saturated fat, and added sugars are associated with reduced risk for prediabetes and T2D [10,13,14,15]. In middle-aged and older adults, adherence to healthy eating patterns such as the Mediterranean diet, Dietary Approaches to Stop Hypertension (DASH) diet, and federal dietary recommendations reduces the risk for T2D [13,14,16]. The Dietary Inflammatory Index (DII), an alternative dietary pattern that quantifies the inflammatory effects of dietary intake, is linked with prediabetes and T2D, where more pro-inflammatory diets are associated with increased risk [17,18]. However, most studies evaluating the relationship between diet and T2D risk have been conducted in middle-aged or older adults or only incidentally included young adults [11,19,20]. Less is understood about the impact of diet quality or dietary changes on T2D risk in young adulthood.

Few prospective studies have examined the relationships between the DASH diet, Mediterranean diet, or other dietary patterns and T2D in youth [21,22,23]. Findings in children and adolescents suggest that increased adherence to the DASH diet may improve cardiovascular and metabolic risk factors [21] and that weight control is critical in T2D prevention [22,24]. Limited studies exist on the development of T2D in young adults [25,26,27,28] though this life stage may represent a critical window for behavior change and diabetes prevention, as young people transition from their adolescent years into independent adulthood [29].

The purpose of this study was to examine the relationship between diet quality and risk for T2D in a cohort of primarily Hispanic young adults. Participants were evaluated for glucose dysregulation and diet quality at age 17–22 and again after approximately four years. Glucose regulation was assessed using hemoglobin A1c (HbA1c) and 2-h oral glucose tolerance tests (OGTTs). We hypothesize that higher diet quality will be protective against glucose dysregulation and that improvement in diet quality between visits will reduce the risk for prediabetes and type 2 diabetes.

## 2. Materials and Methods

### 2.1. Cohort

Between 2014 and 2018, a subset of 158 participants between 17 and 22 years old were recruited from the Children’s Health Study (CHS) [30] for the Meta-AIR study [31]. Subjects were selected if they had overweight or obesity in early adolescence, had not been diagnosed with type 1 or type 2 diabetes, had no medical conditions, and were taking no medications that affect glucose metabolism [31]. Between January 2020 and March 2022, 140 of these participants were invited to participate in a follow-up visit. All but 7 participants underwent follow-up testing during the COVID-19 pandemic. All study visits were completed at the Diabetes and Obesity Research Institute at the University of Southern California. This study was approved by the University of Southern California Institutional Review Board. Written informed consent was obtained from participants at both baseline and follow-up visits or by participants and their guardians for those under age 18 at baseline.

Of the 158 participants at baseline, 155 had diet data and data for at least one outcome. Eighty-six of these participated in the follow-up (Figure S1). An additional three CHS participants without baseline data completed the follow-up visit.

### 2.2. Glucose Outcomes

A 2-h oral glucose tolerance test (OGTT) was performed at each visit, using a glucose load of 1.75 g of glucose per kg of body mass (max 75 g). Blood was sampled before the glucose challenge and at 30-, 60-, 90- and 120-min post-challenge. Glucose concentrations at each time point were measured in plasma. Fasting glucose was also measured using a glucometer before the glucose challenge, and the OGTT was not completed if participants had a fasting value greater than 126 mg/dL. Hemoglobin A1c (HbA1c) was measured in fasting whole blood samples. Glucose area under the curve (AUC) was calculated from the five glucose measurements using the trapezoidal method [32].

Prediabetes and type 2 diabetes were based on clinical cutoffs for HbA1c, fasting plasma glucose, or 2-h plasma glucose [33]. Participants having either HbA1c values of 6.5% or higher, fasting glucose of 126 mg/dL or higher, or 2-h glucose of 200 mg/dL or higher were considered to have type 2 diabetes, while those with HbA1c between 5.7% and 6.4%, fasting glucose between 100 mg/dL and 125 mg/dL, or 2-h glucose between 140 mg/dL and 199 mg/dL were categorized as prediabetic.

### 2.3. Adiposity Outcomes

Weight and height were measured at each visit, and BMI calculated as kg per meter squared (kg/m^2^). Body composition was assessed using dual-energy X-ray absorptiometry (DEXA) whole body scans. Baseline scans were performed on either a Hologic QDR 4500W or Horizon W machine at baseline, while all follow-up scans were performed on the Horizon W. Body composition measures included total body fat percentage, fat mass to height ratio, fat free mass index (FFMI, kg/m^2^), android to gynoid ratio, trunk to leg ratio, trunk to limb ratio, and visceral adipose tissue (VAT, in^3^). Only body fat percentage and fat mass to height ratio were measured on the QDR 4500W machine.

### 2.4. Diet Assessment

At each visit, participants were asked to complete two 24-h dietary recalls on non-consecutive days: one weekday and one weekend day. Baseline recalls were completed by trained interviewers using the Nutritional Data System for Research (NDSR) software version 2014, developed by the Nutrition Coordinating Center (University of Minnesota, Minneapolis, MN, USA) [34], while follow-up recalls used the Automated Self-Administered 24-h (ASA24) Dietary Assessment Tool, version (2018), developed by the National Cancer Institute, Bethesda, MD, USA [35]. An average of the values from both days was calculated for each diet component. At baseline, 16 participants (10.3%) completed only one recall, and 9 (10.2%) completed only one recall at follow-up. If a participant did not complete both recalls, values from the single recall were used.

Four diet indices were calculated from the recall data at both the baseline and follow-up visits: the 2015 Healthy Eating Index (HEI), DASH score, Mediterranean Diet Score (MDS), and DII. The HEI ranges from 0–100, is based on adherence to the United States Department of Agriculture (USDA) 2015 Dietary Guidelines [36] and contains the following thirteen elements standardized to calorie intake: total fruit, whole fruit, total vegetables, greens and beans, whole grains, refined grains, dairy, total protein foods, seafood and plant proteins, mono- and polyunsaturated fatty acids, saturated fats, sodium, and added sugars. The DASH scoring method follows the calculation proposed by Mellen et al. [37], using nutrient goals for DASH diet adherence. This DASH score ranges from 0 to 8 and includes the following elements standardized to calorie intake: protein, fiber, magnesium, calcium, potassium, total fat, saturated fat, cholesterol, and sodium. One point was assigned if the nutrient goal was met, and half of a point was assigned if an intermediate nutrient goal was met. The MDS was calculated using the method developed by Trichopoulou et al. [38], which ranges from 0 to 9 with ten components: vegetables, legumes, fruits and nuts, dairy, cereals, meat, poultry, fish, alcohol, and ratio of mono- to saturated fats. For each component, one point was assigned for exceeding the sex-specific median. The DII was adapted from Shivappa et al. [39], with negative values indicating an anti-inflammatory diet and positive values indicating a pro-inflammatory diet. For each element, a centered percentile was calculated by comparing the reported intake to a global mean and standard deviation of intake. This centered percentile was multiplied by the element’s overall inflammatory effect score, and the scores for all elements were summed to produce the DII score. Twenty-eight of the forty-five elements from Shivappa et al. [39] were included: alcohol, beta-carotene, caffeine, carbohydrates, cholesterol, calories, total fat, fiber, iron, magnesium, folic acid, mono- and polyunsaturated fatty acids, omega-3 and omega-6 fatty acids, protein, saturated fat, selenium, zinc, and vitamins A, B1 (thiamin), B2 (riboflavin), B3 (niacin), C, D, and E. The remaining elements were excluded because they are not specifically captured by the recalls systems used in this study. Trans fats were banned by the United States Food and Drug Administration in 2015, with a 2018 deadline for implementation [40], and were excluded from the DII calculation in the follow-up visit.

### 2.5. Covariates

Questionnaires were administered to collect sociodemographic information, including age, sex, race and ethnicity, physical activity, and parental education. Ethnicity was categorized as non-Hispanic White, Hispanic, or Other. Parental education was categorized as “Did not complete high school”, “Completed high school”, “Completed more than high school”, or “Don’t know”. At baseline, physical activity was assessed as a binary variable, where participants responded yes or no to the question “Do you exercise?”. At follow-up, physical activity was assessed using the International Physical Activity Questionnaire Short Form [41], and metabolic equivalent of task (MET) minutes were calculated according to the scoring guidelines. Participants were considered to have “high” physical activity if they met either of the following criteria: (1) reported vigorous physical activity (VPA) three or more days per week and 1500 or more MET min per week or (2) seven days of any combination of VPA, moderate physical activity (MPA), and walking for at least 3000 MET min. Participants were considered to have “moderate” physical activity if they (1) reported at least 3 days of VPA where the activity lasted at least 30 min or (2) five or more days of MPA or walking where the activity lasted at least 30 min or (3) five or more days of some combination of VPA, MPA, and walking for at least 600 MET min. Participants were categorized as having “low” physical activity if they did not meet any of these criteria.

### 2.6. Statistical Analysis

Descriptive statistics were calculated for all outcomes and exposures. Pearson’s correlations were calculated between the four diet scores at each visit separately and between time points. Independent two-sample *t*-tests, chi-square tests, or Fisher’s exact tests were used to test for differences in participant demographics between the baseline cohort and follow-up cohort. Paired *t*-tests or McNemar–Bowker tests were used to test for differences in exposures and outcomes between visits. Due to the small numbers of participants with values meeting the diagnostic criteria for type 2 diabetes, prediabetes and diabetes were combined into one category (prediabetes/T2D) for analysis. Primary outcomes of interest were those related to glucose regulation: prediabetes/T2D, fasting glucose, 2-h glucose, glucose AUC, and HbA1c. Body composition measurements were secondary outcomes: BMI, body fat percent, FFMI, fat mass to height ratio, android to gynoid ratio, trunk to leg ratio, trunk to limb ratio, and VAT.

Cross-sectional analyses were performed for both baseline and follow-up visits, using multivariable linear regression for continuous outcomes and logistic regression for prediabetes/T2D. For longitudinal analyses, change in diet indices from baseline to follow-up was modeled against change in outcome using linear regression for continuous outcomes, or against diabetes at follow-up using logistic regression. Longitudinal models also adjusted for baseline diet score. Beta coefficients for exposures were scaled to one standard deviation (SD) of the exposure to account for the differing scales.

All analyses included the following covariates: age, ethnicity, physical activity, energy intake, and parental education. Because these factors were not accounted for in the scoring system, analyses with HEI, DASH, and DII scores additionally controlled for sex, and analyses with MDS additionally controlled for energy intake. BMI and body fat percent were presumed to be on the causal pathway between diet and prediabetes and T2D and were not included as covariates in the main analyses to avoid overadjustment [42].

### 2.7. Sensitivity Analyses

For all diet indices and glucose outcomes, two additional analyses were performed. The first did not include physical activity in as a covariate to determine if it had the potential to confound the relationship between diet and glucose regulation and if it was necessary to control for this variable in the main analysis. The second analysis controlled for body fat percent. Though we expect that body fat (or BMI) is on the causal pathway between diet and T2D, we included it as a covariate to examine the possibility that body fat mediates the relationship between diet and T2D.

We also performed additional logistic regression analyses to examine the association between each adiposity measure and risk for prediabetes/T2D at each visit and to examine the associations between changes in these measures between visits and risk for prediabetes/T2D at the follow-up visit. Models were adjusted for age, sex, ethnicity, parental education, energy intake, and physical activity as in the main analyses.

## 3. Results

Average length of follow-up was 4.1 years (SD = 1.1 years). There were no differences in participant demographics at each visit (Table 1). HEI, DASH, and DII scores significantly decreased from baseline to follow-up (Table 2), and mean fasting glucose and glucose AUC increased (Table 3). Mean BMI and body fat percentage also increased between visits (Table 4).

### 3.1. Prediabetes/T2D

**Figure 1:**
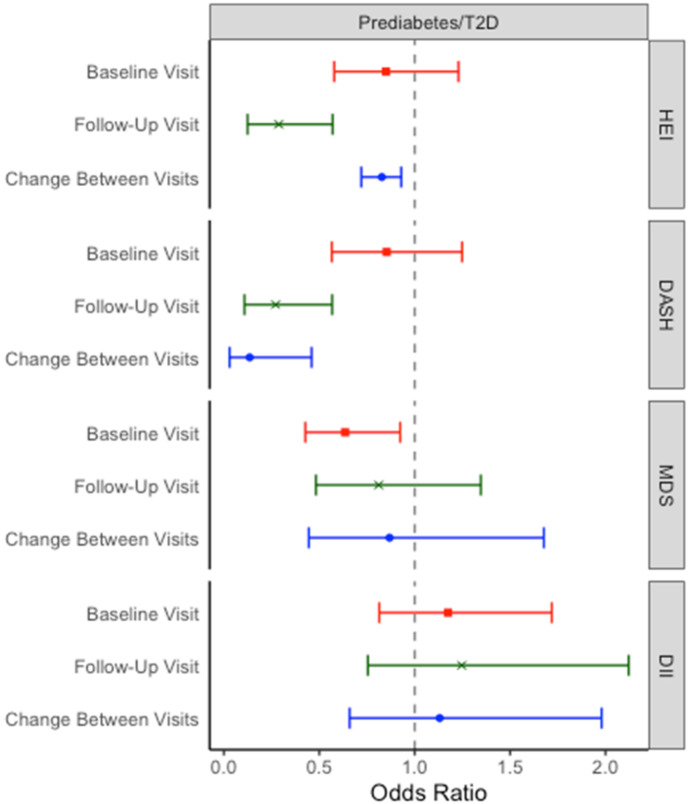
Coefficient plots for the effects of diet quality on prediabetes. “Baseline” and “follow-up” values are the result of cross-sectional analyses of diet quality score and risk of prediabetes/T2D at the same visit. The value for “change between visits” represents the risk of prediabetes/T2D at the follow-up visit associated with change in diet score between the baseline and the follow-up visit. Effects are standardized to one standard deviation of exposure. Covariates: *Baseline and follow*-*up models.* HEI, DASH, and DII models adjusted for age, sex, ethnicity, physical activity, and parental education. MDS models adjusted for energy intake, age, ethnicity, physical activity, and parental education. *Change between visits models.* Baseline and follow-up model covariates + baseline diet score. Abbreviations: DASH: Dietary Approaches to Stop Hypertension; DII: Dietary Inflammatory index; HEI: Healthy Eating Index—2015; MDS: Mediterranean Diet Score.

Positive change in HEI and DASH scores between the baseline and follow-up visits was associated with decreased risk for prediabetes/T2D at follow-up (Figure 1). A one-point increase in DASH score over the follow-up period was associated with a 64% (OR = 0.36, 95% CI: 0.17, 0.68) reduction in risk for prediabetes/T2D at follow-up, while a one-point increase in HEI between visits was associated with a 9% decrease in risk (OR = 0.91, 95% CI: 0.85, 0.96). When scaled by standard deviation of diet index, improvements in DASH diet score reduced the risk for prediabetes/T2D by a greater extent than the HEI (OR = 0.14, 95% CI: 0.03, 0.46; OR = 0.83, 95% CI: 0.72, 0.93, respectively). In the cross-sectional analysis of the follow-up visit, higher HEI and DASH scores were also associated with reduced risk for prediabetes/T2D. At baseline, only MDS was associated with reduced risk for prediabetes/T2D.

### 3.2. Fasting Glucose and Glucose Tolerance

**Figure 2:**
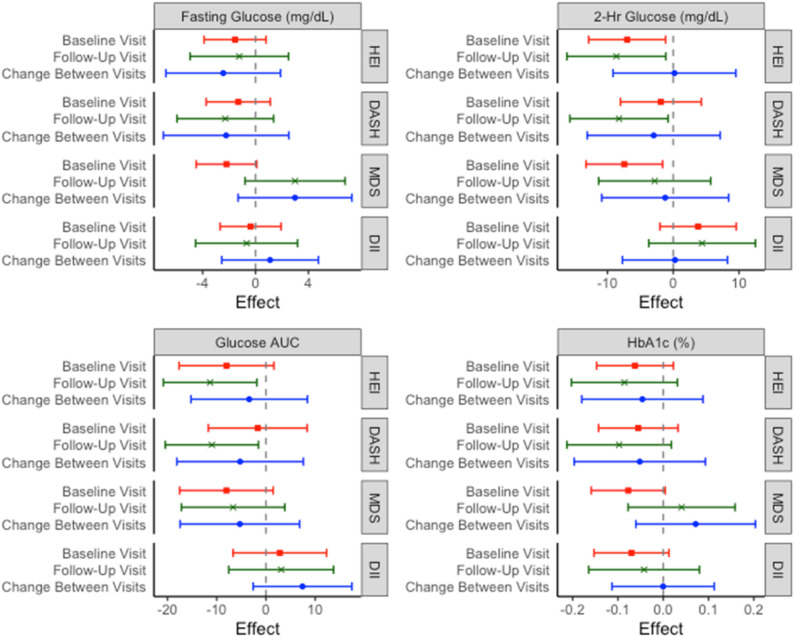
Coefficient plots for the effects of diet quality on glucose measurements. “Baseline” and “follow-up” values are the result of cross-sectional analyses of diet quality score and each outcome. The value for “change between visits” represents the association between the change in diet score between the baseline and the follow-up visit on the change in outcome between visits. Effects are scaled to one standard deviation of exposure. Covariates: *Baseline and follow*-*up models:* HEI, DASH, and DII models adjusted for age, sex, ethnicity, physical activity, and parental education. MDS models adjusted for energy intake, age, ethnicity, physical activity, and parental education. *Change between visits models:* Baseline and follow-up model covariates + baseline diet score. Abbreviations: DASH: Dietary Approaches to Stop Hypertension; DII: Dietary Inflammatory Index; HEI: Healthy Eating Index—2015; MDS: Mediterranean Diet Score.

There were no statistically significant cross-sectional associations between fasting glucose and any dietary index at either visit or between change in diet scores and change in fasting glucose between visits (Figure 2).

Higher HEI scores and higher MDS were associated with lower 2-h glucose values at baseline in the cross-sectional analyses (HEI: β = −7.01, 95% CI: −12.86, −1.16; MDS: β = −7.43, 95% CI: −13.25, −1.61) (Figure 2). Follow-up HEI and DASH scores were inversely associated with 2-h glucose at the same visit (HEI: β = −8.64, 95% CI: −16.16, −1.12; DASH: β = −8.25, 95% CI: −15.71, −0.78) and with glucose AUC (HEI: β = −11.34, 95% CI: −20.84, −1.84; DASH: β = −10.99, 95% CI: −20.44, −1.53).

### 3.3. Hemoglobin A1c

There were no statistically significant associations between HbA1c and any dietary index. However, there were consistent inverse relationships between higher HEI and DASH scores and HbA1c at both visits and between change in HEI or DASH and change in HbA1c between visits although these did not reach the threshold for statistical significance (Figure 2).

### 3.4. Body Composition

The DASH diet was consistently associated with several adiposity measures (Table 5). At the follow-up visit, higher DASH scores were associated with lower BMI (β = −1.64, 95% CI: −3.17, −0.11), body fat percent (β = −1.79, 95% CI: −3.01, −0.57), and fat mass to height ratio (β = −1.09, 95% CI: −3.27, −0.61) at the same visit, and increases in DASH between visits were also inversely associated with change in BMI (β = −1.64, 95% CI: −2.92, −0.36) and body fat percent (β = −1.62, 95% CI: −2.02, −0.17). Similar inverse associations were observed between DASH and measures of central adiposity, including trunk to limb ratio and VAT.

The DII was positively associated with body fat percent in the cross-sectional baseline analyses (Table 5). Though not statistically significant, the DII was also positively associated with most adiposity measurements at both visits, and positive change in DII was associated with positive changes in adiposity from baseline to follow-up.

### 3.5. Sensitivity Analyses

Results from the sensitivity analyses are reported in Supplemental Tables S1–S3. Models that did not adjust for physical activity had slightly larger effect estimates for the relationship between HEI and DASH and impaired glucose tolerance compared to models that did adjust for physical activity. There was little effect on risk for prediabetes/T2D, and the main findings were the same in the physical activity-adjusted and -unadjusted models. Adjustment for body fat percent also had little effect on the relationships between HEI or DASH and prediabetes/T2D, suggesting that it may not mediate the relationship between diet and prediabetes/T2D. However, in most cases, controlling for body fat percent attenuated the effects of each diet on all other glucose outcomes.

BMI, body fat percent, FFMI, fat mass to height ratio, and VAT were significantly associated with increased risk for prediabetes/T2D at all time points (Table S4). At the follow-up visit only, android to gynoid ratio, trunk to leg ratio, and trunk to limb ratio were also positively associated with prediabetes/T2D.

## 4. Discussion

We observed strong inverse associations both in cross-sectional and longitudinal analyses between the HEI and DASH diet and risk of prediabetes/T2D. We also found negative associations between the HEI and DASH diet and 2-h glucose, HbA1c, fasting glucose, and glucose AUC at both visits and in the longitudinal analysis though these relationships were not all statistically significant. The MDS was not consistently associated with prediabetes/T2D, glucose measurements, or body composition. We also observed inverse relationships between HEI, DASH, and MDS with measures of adiposity and body composition, suggesting that high diet quality may be protective against obesity and adverse accumulation of adipose tissue. The period between late adolescence to early adulthood is one of transition, where young people begin to live independently and gain more control of their lifestyles. However, there are limited assessments of change in diet quality during this transition [43], and these results emphasize the importance of considering diet quality in T2D risk within this age group.

To our knowledge, no other study has evaluated the longitudinal relationship between glucose dysregulation and HEI, DASH, MDS, and DII in young adults. Several meta-analyses have summarized the relationship between diet quality and type 2 diabetes, prediabetes, or other measures of glucose dysregulation in older adults. These analyses consistently report strong protective effects of healthy dietary patterns, including the DASH and HEI [10,13,15]. However, previous reviews found effects of similar magnitude between the HEI, DASH, and MDS [14], whereas we report a larger protective effect associated with increases in DASH diet adherence across both visits compared to either the HEI or MDS. The DII has been inconsistently associated with risk of T2D in older adults [17,18] though inflammation is involved in the pathogenesis of type 2 diabetes [44]. Like Vahid (2017), we observed positive associations between DII and impaired glucose intolerance and prediabetes.

Diet is also a risk factor for obesity, which is itself a significant driver of the T2D epidemic in both adults and youth [6,45,46], and increases in body fat greatly increase the risk for future diabetes [47]. Accumulation of visceral fat is also linked to T2D development and severity [48,49]. Our study found similar effects, with multiple adiposity indices significantly associated with increased risk of prediabetes/T2D. Our findings also suggest an inverse relationship between high diet quality and central obesity, with HEI and DASH consistently associated with android to gynoid fat ratio, trunk to limb fat ratios, and VAT. There also appeared to be positive associations between DII and adiposity and visceral fat measures. These findings suggest that high quality diets may reduce the risk of type 2 diabetes in part by reducing total body and visceral fat.

This study has several strengths. Participants were recruited from the Southern California Children’s Health Study [30], which allowed detailed measures of glucose metabolism, diet, body composition, and lifestyle factors. OGTT and DEXA provide highly detailed information about glucose metabolism and body composition, respectively, beyond that of fasting plasma glucose, HbA1c, or BMI alone [50,51]. 2-h glucose and glucose AUC, for example, assess glucose tolerance, and impaired glucose tolerance is an early sign of glucose dysregulation and type 2 diabetes risk not often captured in clinical settings [52]. Additionally, exposures and outcomes were assessed at both visits, which allowed us to examine associations across time. Despite this, we note some limitations. Two systems were used to collect 24-h dietary recalls: the NDSR at baseline and the ASA24 at follow-up. We are not aware of any evidence that this difference would introduce bias away from the null, and any misclassification of diet is expected to be nondifferential and independent of prediabetes/T2D status. It is also common for studies involving multiple cohorts to integrate different diet assessment measures [53,54]. There is a possibility that residual confounding contributed to our reported effects; family history of T2D, maternal obesity, and low birthweight are also associated with young-onset T2D though they are less likely to be associated with diet. However, the magnitude of the relationships we report are large, and any confounding by these or other factors are unlikely to account for the entire effect. Additionally, our sample size for the longitudinal analysis was 85, limiting the statistical power to detect significant relationships. Limitations of one of the DEXA machines used at baseline also limited the available sample size for some adiposity measurements (e.g., android to gynoid fat ratio, trunk to limb fat ratio). However, power was sufficient to identify strong, statistically significant, protective effects of high-quality diets on prediabetes risk.

The COVID-19 pandemic may also have affected our recruitment efforts for the follow-up visit. Our recruitment began as the SARS-CoV-2 virus (COVID-19) was declared first a Public Health Emergency and then a pandemic [55]. The resulting disruptions to daily life would have affected our participants and likely impacted lifestyle factors such as physical activity, sleep, and eating habits as well as stress, social supports, and physical health, all of which may affect non-communicable disease risk [55,56,57]. It is possible that the observed decreases in diet quality between the baseline and follow-up visits may be, in part, due to the pandemic. Even if some of the change in diet were due to changes in lifestyle associated with the COVID-19 pandemic, our findings emphasize the importance of maintaining a healthy diet to reduce the risk for T2D.

Our results indicate that improvements in adherence to the HEI and DASH dietary patterns may reduce risk for T2D. Though both measure diet quality, the construction of each index emphasizes different nutrients and food groups, and there are several ways in which an individual may improve their score and overall diet quality. For example, the HEI rewards greater adherence to the USDA Dietary Guidelines for Americans with higher scores on a 100-point scale [36]. To improve a HEI score, one has several options: (1) increase intake of one or several food groups (fruit, vegetables, seafood, etc.) to the levels recommend by the USDA; (2) reduce intake of added sugars and salt as recommended by the USDA; or (3) reduce the proportion of total grains that come from refined sources or increase the proportion of dietary fats that are mono- or polyunsaturated [58]. Similarly, improvements in DASH diet score could be achieved by reducing consumption of saturated fat, cholesterol, or sodium, or by increasing fiber, magnesium, potassium, and calcium intake [37]. By encouraging changes to overall dietary patterns rather than emphasizing specific foods or nutrients (i.e., kilocalories, sugar-sweetened beverages), individuals may have more flexibility in their choice of dietary habits to alter or methods of alteration, leading to more successful behavior change [59,60].

## 5. Conclusions

Late adolescence to early adulthood is a period of significant change and represents an important window in which to establish lifelong habits [29]. To our knowledge, this study is one of few to evaluate the impact of dietary changes on glucose regulation in people between the ages of 18 and 30. We found that adherence to the DASH diet and USDA Dietary Guidelines is associated with reduced risk for prediabetes and better glucose tolerance. Improvement in DASH or HEI scores over the follow-up period was also associated with lower risk for prediabetes or type 2 diabetes, with the strongest effects observed for the DASH diet. These findings indicate that the DASH dietary pattern may be a promising target for diabetes prevention efforts in young adults.

## Figures and Tables

**Table 1:** Descriptive statistics for participant demographics at baseline and follow-up.

	Baseline (*n* = 155)	Follow-Up (*n* = 88) ^1^	Baseline vs. Follow-Up *p*-Value ^2^
Age (years), Mean (SD)	19.7 (1.2)	24.1 (0.8)	-
Sex, *n* (%) Female Male	71 (45.8) 84 (54.2)	46 (52.3) 42 (47.7)	0.40
Ethnicity, *n* (%) Hispanic/Latino Non-Hispanic White Other	94 (60.6) 52 (33.5) 9 (5.8)	50 (56.8) 30 (34.1) 8 (9.1)	0.60
Parental Education, *n* (%) Did not complete high school Completed high school More than high school Don’t know	31 (20.0) 23 (14.8) 96 (61.9) 5 (3.2)	15 (17.0) 12 (13.6) 56 (63.6) 5 (5.7)	0.76
Exercise ^3^, *n* (%) Yes No	118 (76.1) 37 (23.9)	-	-
Physical Activity Category, *n* (%) High Moderate Low Missing, *n* (%)	-	50 (56.8) 21 (23.9) 16 (18.2) 1 (1.1)	-

^1^ Includes three participants who did not complete the baseline visit. ^2^ *p*-values calculated using chi-Square or Fisher’s exact tests. ^3^ Response to the question “Do you exercise?”. SD: standard deviation.

**Table 2:** Descriptive statistics for diet at baseline, follow-up, and change between visits.

	Baseline (*n* = 155)	Follow-Up (*n* = 88)	Change between Baseline and Follow-Up (*n* = 85) ^1^	Baseline vs. Follow-Up *p*-Value ^2^
HEI, Mean (SD) Range: 0–100	52.7 (13.0)	49.7 (12.5)	−4.9 (13.2)	<0.001
MDS, Mean (SD) Range: 0–9	5.03 (1.23)	4.92 (1.53)	−0.22 (1.79)	0.25
DASH, Mean (SD) Range: 0–8	2.26 (1.51)	1.74 (1.31)	−0.45 (1.53)	0.009
DII, Mean (SD)	0.81 (1.56)	0.29 (2.05)	−0.44 (1.98)	0.044
Energy (kcal), Mean (SD)	2053 (630)	2223 (773)	158 (792)	0.070

^1^ Three additional CHS participants participated in the second visit without having completed the first. ^2^ *p*-values calculated using paired *t*-tests. Abbreviations: HEI: Healthy Eating Index—2015; MDS: Mediterranean Diet Score; DASH: Dietary Approaches to Stop Hypertension; DII: Dietary Inflammatory Index.

**Table 3:** Descriptive statistics for glucose outcomes at baseline, follow-up, and change between visits.

	Baseline (*n* = 155)	Follow-Up (*n* = 88)	Change between Baseline and Follow-Up (*n* = 85) ^1^	Baseline vs. Follow-Up *p*-Value ^2^
Fasting Glucose, Mean (SD) Missing: *n* (%)	91. (14) 1 (0.6)	95 (16) 1 (1.1)	5 (15) 1 (1.2%)	0.003
2-h Glucose, Mean (SD) Missing: *n* (%)	123 (37) 1 (0.6)	122 (35) 4 (4.5)	3 (32) 4 (4.7)	0.39
HbA1c, Mean (SD) Missing: *n* (%)	5.25 (0.53) 1 (0.6)	5.26 (0.51)	0.042 (0.46)	0.35
Glucose AUC, Mean (SD) Missing: *n* (%)	267 (59) 1 (0.6)	269 (44) 6 (6.8)	11 (40) 6 (7.1)	0.023
Diabetes, *n* (%)				0.17
No Diabetes	109 (70.3)	54 (61.4)		
Prediabetes	42 (27.1)	30 (34.1)		
Type 2 Diabetes	3 (1.9)	4 (4.5)		
Missing	1 (0.6)			

^1^ Three additional CHS participants participated in the second visit without having completed the first. ^2^ *p*-values calculated using paired *t*-tests for continuous variables and McNemar–Bowker test for diabetes categories. Abbreviations: SD: standard deviation; HbA1c: hemoglobin A1c; AUC: area under the curve.

**Table 4:** Descriptive statistics for body composition at baseline, follow-up, and change between visits.

	Baseline (*n* = 155)	Follow-Up (*n* = 88)	Change between Baseline and Follow-Up (*n* = 85) ^1^	Baseline vs. Follow-Up *p*-Value ^2,3^
BMI Category, *n* (%) Normal Weight Overweight Obese	24 (15.5) 73 (47.1) 58 (37.4)	12 (13.6) 34 (38.6) 42 (47.7)		0.47
BMI (kg/m^2^), Mean (SD)	29.9 (5.1)	31.7 (7.0)	1.8 (4.3)	<0.001
Body Fat %, Mean (SD) Missing: *n* (%)	34.8 (8.6) -	38.5 (8.3) 2 (2.3)	3.1 (4.7) 2 (2.4)	<0.001
FFMI (kg/m^2^), Mean (SD) Missing: *n* (%)	18.5 (2.5) -	17.7 (2.9) 2 (2.3)	−0.6 (1.5) 2 (2.4)	0.001
Fat Mass:Height Ratio, Mean (SD) Missing: *n* (%)	10.8 (4.3) 98 (63.2)	12.2 (4.7) 2 (2.3)	1.6 (2.1) 47 (55.3)	<0.001
Android:Gynoid Ratio, Mean (SD) Missing: *n* (%)	(0.14) 98 (63.2)	1.01 (0.15) 2 (2.3)	0.015 (0.085) 47 (55.3)	0.30
Trunk:Leg Ratio, Mean (SD) Missing: *n* (%)	0.95 (0.13) 98 (63.3)	0.97 (0.13) 2 (2.3)	0.016 (0.077) 47 (55.3)	0.20
Trunk:Limb Ratio, Mean (SD) Missing: *n* (%)	1.05 (0.20) 98 (63.3)	1.10 (0.23) 2 (2.3)	0.051 (0.11) 47 (55.3)	0.005
VAT Volume (in^3^), Mean (SD) Missing: *n* (%)	592 (301) 98 (63.3)	633 (325) 2 (2.3)	88 (148) 47 (55.3)	<0.001

^1^ Three additional CHS participants participated in the second visit without having completed the first. ^2^ *p*-values calculated using *t*-tests for continuous variables and McNemar–Bowker test for BMI category. ^3^ Fifty-seven participants completed the DEXA scan on a machine that provided additional body composition indices. Abbreviations: BMI, body mass index; FFMI, fat free mass index; VAT, visceral adipose tissue; SD, standard deviation.

**Table 5:** Estimated effect size and 95% CI for the effect of 1 standard deviation increase in diet score on body composition.

Diet	Outcome	Effect Estimate, β (95% CI)		
		Baseline ^1^	Follow-Up ^1^	Change between Visits ^2^
Healthy Eating Index—2015 (HEI)				
BMI (kg/m^2^)		−0.62 (−1.45, 0.21)	−1.33 (−2.89, 0.24)	−0.38 (−1.62, 0.85)
Body Fat (%)		−0.85 (−1.86, 0.16)	−1.09 (−2.37, 0.18)	0.40 (−0.92, 1.73)
FFMI (kg/m^2^)		−0.14 (−0.46, 0.17)	−0.46 (−1.04, 0.12)	−0.23 (−0.64, 0.18)
Fat Mass:Height Ratio		−0.56 (−1.74, 0.62)	−0.73 (−1.68, 0.22)	−0.36 (−1.50, 0.78)
Android:Gynoid Ratio		−0.045 (−0.087, −0.0036)	−0.043 (−0.071, −0.014)	−0.014 (−0.061, 0.034)
Trunk:Leg Ratio		−0.040 (−0.077, −0.0028)	−0.035 (−0.060, −0.0087)	−0.0013 (−0.043, 0.041)
Trunk:Limb Ratio		−0.052 (−0.11, 0.010)	−0.052 (−0.099, −0.0048)	−0.036 (−0.092, 0.020)
VAT (in^3^)		−65.78 (−161.45, 29.49)	−60.54 (−132.21, 11.13)	−48.05 (−123.33, 27.23)
Dietary Approaches to Stop Hypertension (DASH) Score				
BMI (kg/m^2^)		0.067 (−0.80, 0.94)	−1.64 (−3.17, −0.11)	−1.63 (−2.91, −0.35)
Body Fat (%)		0.12 (−0.94, 1.18)	−1.79 (−3.01, −0.57)	−1.61 (−3.01, −0.21)
FFMI (kg/m^2^)		−0.036 (−0.36, 0.29)	−0.49 (−1.06, 0.088)	−0.41 (−0.85, 0.024)
Fat Mass:Height Ratio		0.50 (−0.89, 1.88)	−1.09 (−2.02, −0.17)	−1.50 (−2.73, −0.27)
Android:Gynoid Ratio		−0.015 (−0.066, 0.035)	−0.043 (−0.071, −0.015)	−0.047 (−0.098, 0.0045)
Trunk:Leg Ratio		−0.023 (−0.068, 0.022)	−0.039 (−0.064, −0.014)	−0.037 (−0.084, 0.0097)
Trunk:Limb Ratio		−0.018 (−0.093, 0.057)	−0.052 (−0.099, −0.0057)	−0.073 (−0.13, −0.011)
VAT (in^3^)		42.25 (−70.97, 155.46)	−76.57 (−146.46, −6.68)	−100.39 (−183.62, −17.17)
Mediterranean Diet Score (MDS)				
BMI (kg/m^2^)		−0.090 (−0.91, 0.73)	−0.71 (−2.28, 0.86)	0.27 (−0.95, 1.49)
Body Fat (%)		−0.45 (−1.69, 0.79)	−0.48 (−2.35, 1.39)	1.24 (−0.062, 2.55)
FFMI (kg/m^2^)		0.078 (−0.32, 0.47)	0.075 (−0.57, 0.72)	−0.00040 (−0.42, 0.42)
Fat Mass:Height Ratio		−0.37 (−1.49, 0.75)	−0.28 (−1.38, 0.83)	−0.081 (−1.11, 0.95)
Android:Gynoid Ratio		0.00054 (−0.042, 0.043)	−0.0049 (−0.039, 0.030)	0.021 (−0.015, 0.057)
Trunk:Leg Ratio		−0.030 (−0.065, 0.0037)	−0.0042 (−0.035, 0.027)	−0.0030 (−0.041, 0.035)
Trunk:Limb Ratio		−0.044 (−0.10, 0.014)	−0.0073 (−0.062, 0.047)	−0.011 (−0.064, 0.042)
VAT (in^3^)		−21.86 (−109.41, 65.68)	−17.16 (−92.10, 57.79)	−25.82 (−98.45, 46.81)
Dietary Inflammatory Index (DII)				
BMI (kg/m^2^)		0.86 (0.044, 1.67)	−0.67 (−2.32, 0.97)	−0.21 (−1.24, 0.83)
Body Fat (%)		2.04 (1.09, 2.99)	1.13 (−0.19, 2.45)	0.44 (−0.66, 1.54)
FFMI (kg/m^2^)		−0.073 (−0.38, 0.23)	−0.60 (−1.20, −0.0068)	−0.16 (−0.50, 0.18)
Fat Mass:Height Ratio		0.88 (−0.23, 1.99)	−0.17 (−1.17, 0.84)	0.52 (−0.33, 1.37)
Android:Gynoid Ratio		0.031 (−0.010, 0.072)	0.014 (−0.017, 0.045)	0.035 (0.0025, 0.068)
Trunk:Leg Ratio		0.027 (−0.010, 0.063)	0.021 (−0.0070, 0.048)	0.017 (−0.014, 0.048)
Trunk:Limb Ratio		0.028 (−0.033, 0.089)	0.023 (−0.027, 0.074)	0.029 (−0.014, 0.071)
VAT (in^3^)		47.00 (−44.96, 138.95)	−22.50 (−97.94, 52.94)	17.77 (−42.53, 78.08)

^1^ Model A: outcome ~ diet score + covariates. ^2^ Model B: Δoutcome ~ Δdiet score + covariates. Model A covariates: HEI, DASH, and DII models adjusted for age, sex, ethnicity, physical activity, and parental education. MDS models adjusted for energy intake, age, ethnicity, physical activity, and parental education. Model B covariates: Model A covariates + baseline diet score. Effects were scaled to 1 standard deviation of exposure. Abbreviations: BMI: body mass index; FFMI: fat-free mass index; VAT: visceral adipose tissue.

## Data Availability

The data presented in this study are available on request from the corresponding author. The data are not publicly available to protect participants’ identifiable information.

## Supplementary Materials

The following supporting information can be downloaded at: https://www.mdpi.com/article/10.3390/nu14183734/s1, Figure S1: Flowchart for study recruitment; Table S1: Results (effects and 95% CIs) for sensitivity analyses at the baseline visit; Table S2: Results (effects and 95% CIs) for sensitivity analyses at the follow-up visit; Table S3: Results (effects and 95% CIs) for sensitivity analyses for the effects of change in diet score between visits; Table S4: Estimated effect size and 95% CI for the relationship between body composition and risk for prediabetes/type 2 diabetes).
