# Supplementary material for: Diet Quality Is Associated with Glucose Regulation in a Cohort of Young Adults
Source: Nutrients. 2022 Sep 10;14(18):3734. doi: 10.3390/nu14183734 (PMC9501084; doi:10.3390/nu14183734)
Supplement: Supplementary file 1 [file nutrients-14-03734-s001.zip › nutrients-1896397-supplementary.pdf]

Supplemental Tables and Figures

Figure S1. Flowchart for study recruitment.

Table S1. Results (effects and 95% CIs) for sensitivity analyses at the baseline visit.

Table S2. Results (effects and 95% CIs) for sensitivity analyses at the follow up visit.

Table S3. Results (effects and 95% CIs) for sensitivity analyses for the effects of change in diet score between visits

Table S4. Estimated effect size and 95% CI for the relationship between body composition and risk for prediabetes/type 2 diabetes.

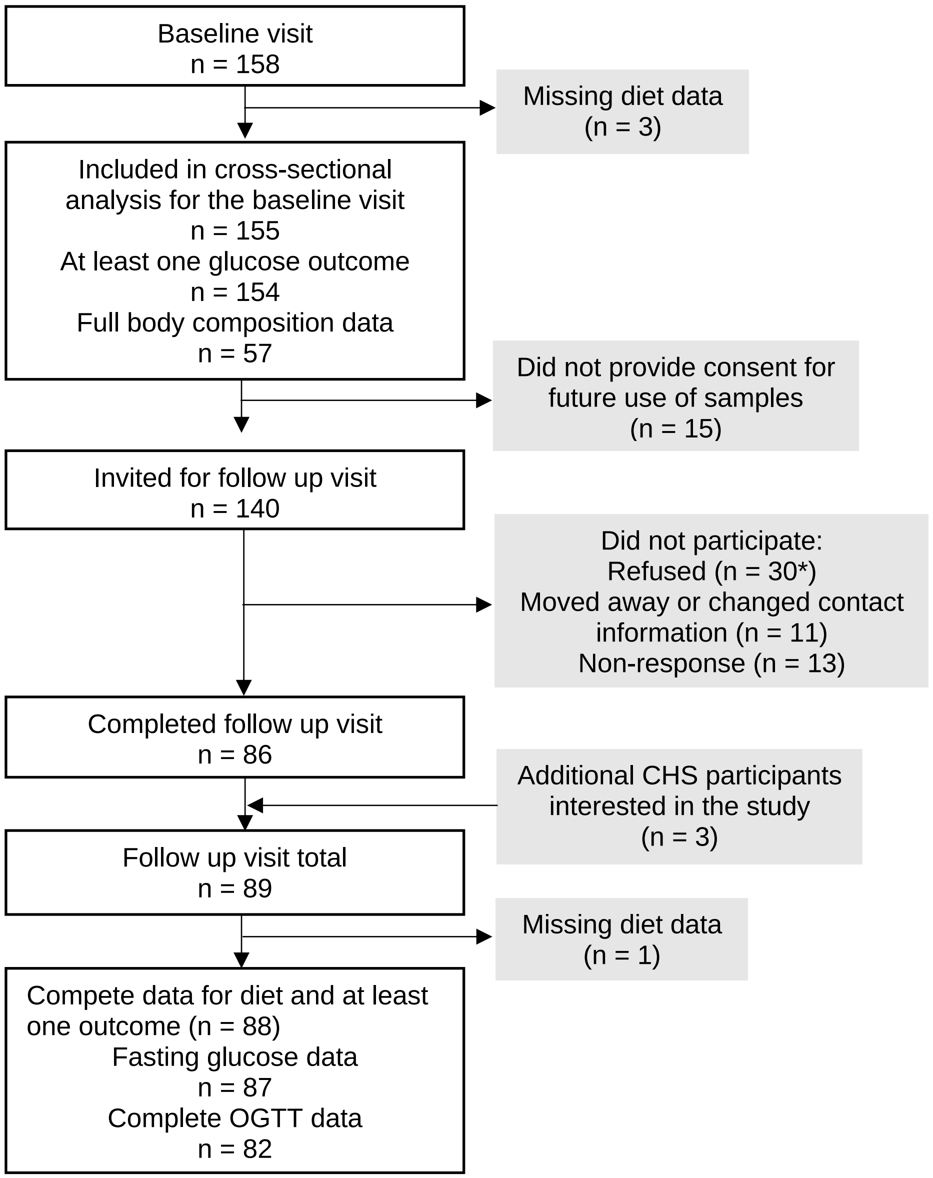

Figure S1. Flowchart for study recruitment.
*Includes those who refused for reasons related to COVID-19 and no-shows for scheduled visits.

Table S1. Results (effects and 95% CIs) for sensitivity analyses at the baseline visit.

	HEI	DASH	MDS	DII
Outcome	Effect (95% CI)	Effect (95% CI)	Effect (95% CI)	Effect (95% CI)
Fasting glucose				
Model 1	-1.52 (-3.84, 0.80)	-1.53 (-3.90, 0.85)	-1.90 (-4.18, 0.39)	0.089 (-2.59, 2.77)
Model 2	-1.55 (-3.89, 0.79)	-1.31 (-3.73, 1.11)	-2.20 (-4.48, 0.093)	-0.37 (-2.68, 1.93)
Model 3	-1.41 (-3.78, 0.96)	-1.33 (-3.75, 1.10)	-2.12 (-4.42, 0.18)	-0.83 (-3.27, 1.61)
2-hour glucose				
Model 1	-**7.26 (-13.04, -1.49)**	-2.62 (-8.63, 3.39)	**-6.90 (-12.62, -1.18)**	4.36 (-2.36, 11.08)
Model 2	**-7.01 (-12.86, -1.16)**	-1.88 (-8.03, 4.28)	**-7.43 (-13.25, -1.61)**	3.78 (-2.02, 9.58)
Model 3	**-5.73 (-11.51, 0.052)**	-2.00 (-7.97, 3.97)	**-6.69 (-12.31, -1.08)**	0.90 (-5.09, 6.90)
HbA1c				
Model 1	-0.063 (-0.15, 0.021)	-0.065 (-0.15, 0.021)	-0.066 (-1.47, 0.016)	-0.024 (-0.12, 0.072)
Model 2	-0.062 (-0.15, 0.022)	-0.055 (-0.14, 0.033)	-0.08 (-0.16, 0.004)	-0.070 (-0.15, 0.013)
Model 3	-0.063 (-0.15, 0.023)	-0.055 (-0.14, 0.033)	-0.075 (-0.16, 0.007)	-0.081 (-0.17, 0.007)
Glucose AUC				
Model 1	-8.57 (-18.29, 1.15)	-3.06 (-13.08, 6.96)	-7.39 (-17.02, 2.25)	2.43 (-8.82, 13.69)
Model 2	-8.02 (-17.65, 1.61)	-1.67 (-11.71, 8.37)	-8.03 (-17.53, 1.46)	2.81 (-6.69, 12.31)
Model 3	-6.38 (-16.00, 3.24)	-1.83 (-11.71, 8.05)	-7.28 (-16.69, 2.12)	-1.06 (-11.0, 8.85)
T2D/prediabetes				
Model 1	0.83 (0.57, 1.19)	0.84 (0.56, 1.21)	**0.64 (0.44, 0.93)**	1.28 (0.84, 2.00)
Model 2	0.85 (0.58, 1.23)	0.85 (0.57, 1.25)	**0.64 (0.43, 0.92)**	1.17 (0.81, 1.72)
Model 3	0.89 (0.60, 1.30)	0.84 (0.56, 1.24)	**0.65 (0.44, 0.95)**	1.05 (0.70, 1.57)

Model 1 covariates: age, sex, ethnicity, parental education.

Model 2 covariates: Model 1 + physical activity

Model 3 covariates: Model 2 + body fat percent

MDS models additionally control for baseline energy intake and not for sex. All effects are scaled to 1 SD of exposure.

Table S2. Results (effects and 95% CIs) for sensitivity analyses at the follow up visit.

	HEI	DASH	MDS	DII
Outcome	Effect (95% CI)	Effect (95% CI)	Effect (95% CI)	Effect (95% CI)
Fasting glucose				
Model 1	-0.95 (-4.57, 2.66)	-1.94 (-5.47, 1.60)	2.52 (-1.05, 6.09)	-0.87 (-4.61, 2.86)
Model 2	-1.23 (-4.95, 2.50)	-2.29 (-5.94, 1.37)	2.99 (-0.79, 6.77)	-0.68 (-4.53, 3.18)
Model 3	-0.21 (-3.85, 3.43)	-0.69 (-4.40, 3.03)	3.75 (-0.17, 7.68)	-2.08 (-5.85, 1.69)
2-hour glucose				
Model 1	**-9.87 (-17.35, -2.38)**	**-9.37 (-16.78, -1.95)**	-1.03 (-9.23, 7.16)	5.74 (-2.31, 13.80)
Model 2	**-8.64 (-16.16, -1.12)**	**-8.25 (-15.71, -0.78)**	-2.82 (-11.33, 5.70)	4.39 (-3.72, 12.50)
Model 3	-5.60 (-12.39, 1.19)	-3.72 (-10.07, 4.04)	-1.01 (-8.78, 6.75)	-0.22 (-7.60, 7.15)
Glucose AUC				
Model 1	**-12.74 (-22.39, -3.10)**	**-12.37 (-22.00, -2.75)**	-3.93 (-14.21, 6.34)	4.37 (-6.50, 15.24)
Model 2	**-11.34 (-20.84, -1.84)**	**-10.99 (-20.44, -1.53)**	-6.67 (-17.17, 3.82)	3.08 (-7.57, 13.73)
Model 3	-7.51 (-16.21, 1.19)	-5.61 (-14.54, 3.32)	-4.24 (-14.22, 5.73)	-1.84 (-11.49, 7.81)
HbA1c				
Model 1	-0.085 (-0.20, 0.028)	-0.098 (-0.21, 0.015)	0.040 (-0.070, 0.15)	-0.038 (-0.16, 0.080)
Model 2	-0.086 (-0.20, 0.031)	-0.10 (-0.21, 0.018)	0.041 (-0.078, 0.16)	-0.042 (-0.16, 0.080)
Model 3	-0.059 (-0.18, 0.058)	-0.055 (-0.17, 0.064)	0.050 (-0.073, 0.17)	-0.075 (-0.20, 0.045)
T2D/prediabetes				
Model 1	**0.28 (0.12, 0.56)**	**0.31 (0.13, 0.61)**	0.76 (0.47, 1.22)	1.31 (0.80, 2.23)
Model 2	**0.29 (0.12, 0.57)**	**0.27 (0.11, 0.57)**	0.81 (0.48, 1.35)	1.25 (0.75, 2.12)
Model 3	**0.19 (0.059, 0.48)**	**0.26 (0.082, 0.67)**	0.80 (0.45, 1.40)	1.01 (0.52, 2.00)

Model 1 covariates: age, sex, ethnicity, parental education

Model 2 covariates: Model 2 + physical activity

Model 3 covariates: Model 1 + body fat percent

MDS models additionally control for baseline energy intake and not for sex. All effects are scaled to 1 SD of exposure.

Table S3. Results (effects and 95% CIs) for sensitivity analyses for the effects of change in diet score between visits

	Δ HEI	Δ DASH	Δ MDS	Δ DII
Outcome	Effect (95% CI)	Effect (95% CI)	Effect (95% CI)	Effect (95% CI)
Δ Fasting glucose				
Model 1	-2.30 (-6.51, 1.90)	-2.01 (-6.57, 2.55)	2.72 (-1.29, 6.73)	0.90 (-2.59, 4.38)
Model 2	-2.44 (-6.78, 1.89)	-2.23 (-6.97, 2.52)	2.98 (-1.32, 7.29)	1.10 (-2.55, 4.76)
Model 3	-2.10 (-6.58, 2.38)	-1.97 (-6.82, 2.89)	3.05 (-1.37, 7.46)	1.12 (-2.47, 4.88)
Δ 2-hour glucose				
Model 1	-0.53 (-9.68, 8.63)	-4.18 (-13.98, 5.62)	0.10 (-8.87, 9.06)	1.00 (-6.72, 8.72)
Model 2	0.18 (-9.15, 9.52)	-2.96 (-13.07, 7.14)	-1.22 (-10.87, 8.43)	0.26 (-7.72, 8.25)
Model 3	0.51 (-9.16, 10.18)	-2.82 (-13.17, 7.53)	-1.41 (-11.31, 8.49)	0.34 (-7.72, 8.41)
Δ Glucose AUC				
Model 1	-4.20 (-15.85, 7.45)	-6.75 (-19.30, 5.81)	-2.57 (-13.97, 8.83)	7.09 (-2.68, 16.86)
Model 2	-3.40 (-15.23, 8.43)	-5.25 (-18.10, 7.59)	-5.30 (-17.44, 6.83)	7.42 (-2.61, 17.45)
Model 3	-2.78 (-15.08, 9.53)	-4.82 (-17.93, 8.29)	-4.55 (-16.97, 7.87)	7.78 (-2.34, 17.89)
Δ HbA1c				
Model 1	-0.049 (-0.18, 0.081)	-0.057 (-0.20, 0.083)	0.065 (-0.057, 0.19)	0.0002 (-0.11, 0.11)
Model 2	-0.046 (-0.18, 0.088)	-0.052 (-0.20, 0.093)	0.072 (-0.060, 0.20)	-0.0003 (-0.11, 0.11)
Model 3	-0.023 (-0.16, 0.11)	-0.035 (-0.18, 0.11)	0.71 (-0.064, 0.21)	0.0051 (-0.11, 0.12)
T2D/prediabetes at the follow up visit				
Model 1	**0.82 (0.72, 0.92)**	**0.15 (0.035, 0.47)**	0.79 (0.42, 1.45)	1.24 (0.74, 2.15)
Model 2	**0.83 (0.72, 0.93)**	**0.14 (0.030, 0.46)**	0.87 (0.45, 1.68)	1.13 (0.66, 1.98)
Model 3	**0.85 (0.74, 0.95)**	**0.15 (0.033, 0.54)**	1.00 (0.51, 1.97)	1.06 (0.60, 1.90)

Model 1 covariates: age, sex, ethnicity, parental education, baseline diet score
Model 2 covariates: Model 2 + physical activity at follow up
Model 3 covariates: Model 1 + body fat percent at baseline
MDS models additionally control for baseline energy intake and not for sex. All effects are scaled to 1 SD of exposure.

Table S4. Estimated effect size and 95% CI for the relationship between body composition and risk for prediabetes/type 2 diabetes.

Exposure	Odds Ratio (95% CI)		
	Baseline[1]	Follow-up[1]	Change between visits[2]
BMI (kg/m^2)	1.11 (1.03, 1.20)	1.17 (1.07, 1.29)	1.19 (1.04, 1.40)
Body Fat (%)	1.067 (1.003, 1.139)	1.41 (1.21, 1.72)	1.26 (1.10, 1.50)
FFMI (kg/m^2)	1.26 (1.04, 1.53)	1.31 (1.06 1.65)	1.53 (1.06, 2.32)
Fat Mass:Height Ratio	1.28 (1.07, 1.59)	1.46 (1.22, 1.82)	2.45 (1.27, 6.57)
Android:Gynoid Ratio[3]	1.057 (1.005, 1.126)	1.10 (1.04, 1.16)	1.15 (0.98, 1.41)
Trunk:Leg Ratio[3]	1.05 (0.99, 1.12)	1.08 (1.03, 1.14)	1.04 (0.87, 1.23)
Trunk:Limb Ratio[3]	1.027 (0.99, 1.07)	1.04 (1.01, 1.07)	1.07 (0.98, 1.18)
VAT[4] (in^3)	1.33 (1.07, 1.74)	1.54 (1.25, 2.00)	2.16 (1.11, 5.21)

Abbreviations: BMI: body mass index; FFMI: fat free mass index; VAT: visceral adipose tissue volume

[1] Model A: outcome ~ diet score + covariates

[2] Model B: Δoutcome ~ Δdiet score + covariates

[3] Effects are expressed per 0.01 unit increase in ratio.

[4] Effects are expressed per 100 in^3 increase in VAT.

Model A covariates: age, sex, ethnicity, parental education, energy intake, and physical activity

Model B covariates: Model A covariates + baseline exposure.
